# Measuring Immersion, Involvement, and Attention Focusing Tendencies in the Mediated Environment: The Applicability of the Immersive Tendencies Questionnaire

**DOI:** 10.3389/fpsyg.2022.931955

**Published:** 2022-07-14

**Authors:** Sándor Rózsa, Rita Hargitai, András Láng, Anikó Osváth, Ernő Hupuczi, István Tamás, János Kállai

**Affiliations:** ^1^Department of Personality and Health Psychology, Institute of Psychology, Károli Gáspár University of the Reformed Church in Hungary, Budapest, Hungary; ^2^Department of Personality and Clinical Psychology, Institute of Psychology, Pázmány Péter Catholic University, Budapest, Hungary; ^3^Institute of Psychology, Art and Science Faculties, University of Pécs, Pécs, Hungary; ^4^Department of Pediatrics, Medical School, University of Pécs, Pécs, Hungary; ^5^Department of Behavioral Sciences, Medical School, University of Pécs, Pécs, Hungary

**Keywords:** absorption, immersion, mental health, Facebook addiction, internet addiction, social adaptation

## Abstract

This study explores the personal predispositions and dependencies while individuals use digital media and communication devices and analyses the statistical features of the Immersive Tendencies Questionnaire (ITQ) that is popular in assessing the personality trait-dependent reaction to mediated environments. The study evaluated 781 healthy graduates and postgraduates, of which 192 were men (average age: 28.6 years) and 589 were women (average age: 28.4 years). We applied several questionnaires to measure immersive tendencies in a mediated environment, adaptive and maladaptive personality predispositions, and problematic Internet use and Facebook addiction scales. We analyze the statistical features of the long and short forms of the Immersive Tendencies Questionnaire. The data obtained support the reliable usage of the short form of the instrument. The factor structure of the questionnaire presents dual facets. First, it indicates an absorptive and immersive tendency in any case of maladaptive tendencies. Second, it reflects an intensive capability to focus on the mediated environment with adequate cognitive control to avoid any contingency of being addicted. The short form of the ITQ is reliable and adequate to assess the relationship between the self-referred and environment-dependent psychological functions.

## Introduction

Immersion is a psychological response to a precisely drawn-up and realistic stimulus set generated by a computer or other medium. Over the last decade, digital media-induced immersion has gained a fundamental place in lifestyle and the conceptual definition of different forms of virtual environments. These mediated environments are used for entertainment, skills training, constructing and maintaining social networks, and other social activities. However, measuring one's capacity to immerse in a mediated environment depends on technical instrumental conditions, personal trait predispositions, and behavioral habits. One method to measure the different sides of an individual's capacity for immersion is the Immersive Tendencies Questionnaire (ITQ) created by Witmer and Singer (1998). This study explores the statistical features of the ITQ and its short form as well as its associations with adaptive and maladaptive personality predispositions.

### Immersion Tendencies Questionnaire

The technology for the construction of mediated environments has improved significantly over the last decade. It has become more realistic and carefully detailed. The immersion experience is far more intense in contemporary times. The tools that are used to measure immersion are examined using virtual reality simulators, social media, and self-reported behavioral habits. Based on Tcha-Tokey et al. (2016), the measuring tools selected included abilities (Computer Self-efficacy Scale, Murphy et al., 1989), presence (Presence Questionnaire, PQ, Kennedy et al., 1993), immersion tendencies Questionnaire, ITQ (Witmer and Singer, 1998), spatial presence (Vorderer et al., 2004), experience and consequences (Simulator Sickness Questionnaire, Bailenson and Yee, 2006), usability (Structure of the System Usability Scale or SUS, Lewis and Sauro, 2009), and emotions (Achievement Emotions Questionnaire, Pekrun et al., 2011). The presence, or the “sense of being there” and immersion concerns “post” or “pre” immersive states. Therefore, it has a multidimensional characteristic. The uncertainty of the assessment is the result of the fluctuating nature of the interaction with the digital environment. Immersion and presence are not continuous and often break owing to fluctuations (Slater and Steed, 2000). Observation of previously mentioned experiences carries great importance in research. From the available tools, only one slide, the ITQ was chosen in this study. The initial version of the scale comprised 29 items. Its factor structure presented a 3-factor solution: *Involvement* examines the level of passive immersion, which can be experienced while reading a book, watching TV, or playing a computer game. *The attention focus* scale deals with the ability to ignore the disturbing effects of the environment. *Commitment to games* involves examining the increased interest in a computer or video game. Based on psychometric analyses, ITQ was reduced to 18 items from the original 29. It measures an individual's level of engagement while being involved in lifelike events, artistic elaborations, experiential actions, and using traditional and/or digital media devices. An immersed individual focuses on the event while also ignoring disturbing conditions in its surroundings. In the immersed state, the individual experiences intense positive and negative emotions, which have consequences for their real life. A high score in the ITQ predicts the intensity of a person's sense of presence as well (Witmer and Singer, 1998). The cluster analysis of the ITQ data revealed three subscales based on 18 items, and the subscale labels are loosely based on the content of the questionnaire items in their cluster.

This study explores the factor structure of ITQ and tests the validity of the factors through immersion-related adaptive and maladaptive personality traits. As the subdimensions of the ITQ have been examined empirically only in a few studies (Robillard et al., 2002; Weibel et al., 2010) (see Table 1). We first analyzed the scale using factor analysis. While selecting personality traits, reliable questionnaires were selected from an immersion-related self-function questionnaire set.

**Table 1:** Overview of the results of the factor analyses of ITQ in international studies.

**References**	**Investigated population**	**Items and structure**	**Statistical method**
Witmer and Singer (1998)	College students *n* = 152	The ITQ was reduced to 18 items from 29. Three-dimensional structure: Involvement, Focus, Games.	Item and cluster analyses
Jerome and Witmer (2002)	College students *n* = 192	29-item version of ITQ. Three-dimensional structure: Involvement, Focus, Games.	SEM Involvement had a high factor weight (0.98), Focus (0.51) and Games (0.22) had low loadings.
Weibel et al. (2010)	Participants *via* an online survey *n* = 220	19-item version of ITQ Factors: Emotional involvement (five items), Absorption (four items).	PCA, Varimax
Robillard et al. (2002)	Virtual reality study *n* = 94	18-item version of ITQ Involvement (five items) Focus (five items) Emotions (four items).	PCA, Varimax

*PCA, Principal component analysis; SEM, Structural equation modeling*.

The Internet and Facebook can be used productively and helpfully for self-development, expanding knowledge, and creating new connections. A virtual reality user can gain reassurance and support through these media outlets, but may also be demotivated, bullied, and offended, much like in real personal connections. Individuals have greater control over their digital presence than over their real appearance, as they can choose to hide non-beneficial aspects of themselves. This representation of an individual is not interactive and does not happen in real-time. Thus, manipulation capabilities are more potent. The main question in this study is whether immersion in the Internet or Facebook is helpful or harmful for a user from the perspectives of social adaptation, everyday wellbeing, and the creation of social networks. The answer is derived by defining self-clarity and comparing seclusion and the need for connection. It is expected that the lack of self-clarification and the immersion capability combined will be connected to problematic Internet and Facebook usage among men and women, and youth and elderly alike.

## Methods

The sample comprised 781 participants, of which 192 were men (M: 28.6, SD: 11.4) and 589 were women (M: 28.4, SD: 11.03). The participants were students at regional universities they had already graduated and were pursuing an additional program for professional training. We gathered data with free access methods. There was no payment made for participation. The participants were informed about the study and asked to indicate their consent in writing following the Helsinki Declaration, ethical approval was sought and received (Document No. 6732 PTE/2017)” which permits the process, the number of the ethical document is No. 6732 PTE/2017. The participants received the questionnaire, which was presented in the below order. Whereas, the questionnaires were filled out anonymously, only gender, age, and education in years were gathered. To inspect adaptation, the Mental Health continuum-short form was chosen, which is a measurement scale that shows the bond between high-quality living standards and levels of adaptation (Mental Health Continuum-Short Form or MHC-SF, Keyes, 2013), the Self-Concept Clarity (SCCS), and Anxiety Sensitivity Index (ASI) were utilized. It measures the level of social adaptation, living standards, and the self-clarity of a person. Two governing aspects were chosen to define social maladaptation and distancing. It was measured by both the Schizotypal Personality Questionnaire-Brief Revisited (SPQ-BR, Cohen et al., 2010) and the Personality Inventory for DSM-5 will be applied for the measurement of psychopathologic vulnerability. The disturbances in adaptability while using digital devices were measured by the Problematic Internet Use Questionnaire (PIUQ, Demetrovics et al., 2008; Koronczai et al., 2011; Király et al., 2015) and Bergen Facebook Addiction Scale (BFAS, Andreassen et al., 2012) was applied. The immersive tendency was defined by the Immersive Tendency Questionnaire (ITQ, Witmer and Singer, 1998), and the Tellegen Absorption Scale (TAS, Tellegen and Atkinson, 1974), which helps measure the intensity of the immersion in the traditional (books and movies) and digital environments. Absorption plays a role in experience involvement and has a strong impact on e-learning competencies in information technological devices (Agarwal and Karahanna, 2000; Saadé and Bahli, 2005; Léger et al., 2014).

### Immersive Tendencies Questionnaire

The Immersive Tendencies Questionnaire (ITQ, Witmer and Singer, 1998) comprises 18 items and utilizes a scale of 1 (never) to 7 (often). It measures the level of immersion in books, movies, and computer games through emotional engagement, the frequency of digital device usage, and the intense attention that leads to the neglect of the current environment. According to Witmer and Singer (1998), the reliability of the scale ranged between 0.75 and 0.81. The scale comprised three subdimensions, namely, involvement (seven items), attentional focus (seven items), and tendency to play video games (two items). Two items (item 9 and item 13) are not included in the subdimensions. These dimensions were based on theoretical considerations; but no statistical procedure was used to confirm the postulated factors (Weibel et al., 2010).

### Tellegen Absorption Scale

Based on Rózsa et al. (2019), the Tellegen Absorption Scale (TAS, Tellegen and Atkinson, 1974; Simor et al., 2011) comprises 34 items and utilizes a scale of 1 (not likely) to 5 (likely). The cumulative results of the TAS represent responsiveness to engaging stimuli, synaesthesia, enhanced cognition, oblivious/dissociative involvement, vivid reminiscence, and enhanced awareness. The cumulative result of the absorption skills is used as an index for self-absorption capability. High values emphasize the willingness to engage in strong self-absorption. Cronbach's alpha for the TAS total score in the current sample was excellent (α = 0.95).

### Self-Concept Clarity Scale

Self-clarity refers to the extent to which an individual's self-beliefs are clearly and confidently defined, internally consistent, and stable. The Self-Concept Clarity Scale (SCCS, Campbell et al., 1996; Hargitai et al., 2020) comprises 12 items and utilizes a Likert scale that ranges from 1 (total refusal—“I do not agree at all”) to 5 (total agreement—“I fully agree”). High values represent a clearer, more stable, and more articulated self-awareness and consistency. However, low values represent a malfunction in beliefs about themselves and uncertainty. The internal consistency of the scale in the current sample was high (α = 0.92).

### Mental Health Continuum Short Form

The Mental Health Continuum Short Form (MHC-SF, Keyes, 2013; Reinhardt et al., 2020) comprises 14 items and measures the emotional, social, and psychological elements of mental health on a scale ranging from 0 (never) to 5 (every day). The cumulative result of this questionnaire shows the level of healthy social adaptation, which reflects a person's positive judgment of their emotional and psychological state. The internal consistency of the scale in the current sample was high (α = 0.89).

### Anxiety Sensitivity Index

The Anxiety Sensitivity Index (ASI, Reiss et al., 1986; Kerekes, 2012) consists of 16 items and utilizes a Likert scale that ranges from 1 (not disturbing) to 5 (very disturbing). The values measure the symptoms of the somatic, cognitive, and social aspects linked to anxiety. The ASI is the most frequently used and reliable tool to measure the tendency to anxiety (Peterson and Plehn, 1999). Empirical researchers use it as a dimension measuring tool. The high scores indicate vulnerability to anxiety (Deacon and Abramowitz, 2006). Cronbach's alpha was 0.91 for this measure in the current sample.

### Schizotypal Personality Questionnaire-Brief Revisited

The Schizotypal Personality Questionnaire-Brief Revisited (SPQ-BR, Cohen et al., 2010; Kállai et al., 2018) is an adequate method to reveal individual vulnerability to schizotypy. Participants use a 5-point Likert scale that ranges from 0 (strongly disagree) to 4 (strongly agree). The SPQ-BR comprises 32 items, including the cognitive, interpersonal, and behavioral components of social adaptation. In this study, the high SPQ-BR total score was accounted for to measure self-boundary weakness and social maladaptation. Cronbach's alpha was excellent (α = 0.92).

### Personality Inventory for DSM-5 (PID-5-BF)

The Personality Inventory for DSM-5 (PID-5-BF, Krueger et al., 2012; Anderson et al., 2014; Birkás et al., 2018) is a 25-item self-reported questionnaire that assesses the five maladaptive personality traits, namely, negative affectivity, detachment, antagonism, disinhibition, and psychoticism. Items are rated on a 4-point Likert Scale (0 = very false or often false to 3 = very true or often true). PID-5-BF generates a score for the overall measure, indicating the rate of social maladaptation. In this study, Cronbach's alpha for total scores was 0.86.

### Bergen Facebook Addiction Scale

The Bergen Facebook Addiction Scale (BFAS, Andreassen et al., 2012; Ferreira da Veiga et al., 2018) comprises 6 self-assuring items with a measurement scale of 1 (very rarely) to 5 (really often). It measures the attribution of significance, mood swings, and the level of tension that comes from withdrawal. The maximum achievable point is 30, and the scale shows the level of addiction to Facebook. The Hungarian version of this questionnaire was translated by a native English speaker and checked by a professional translator who also back-translated it. Cronbach's alpha was 0.81 for this scale in the current sample. To improve the reliability of the questionnaire, a few extra questions were introduced, such as: Do you have a Facebook account? Approximately how many connections do you have on Facebook? How many hours do you spend every day on Facebook on average?

### Problematic Internet Use Questionnaire

The Problematic Internet Use Questionnaire (PIUQ, Demetrovics et al., 2008; Koronczai et al., 2011) consists of 18 items and uses a Likert scale that ranges from 0 (never) to 5 (always). It is based on the three-factor model of Internet addiction, which measures potentially problematic Internet usage. It reveals the compulsive, uncontrollable usage that can lead to the ignorance of social relations. The aggregate high scores of the items indicate maladaptive addiction restricting an individual's emotional and behavioral functions and social adaptive success. The internal consistency of the scale in the current sample was high (α = 0.91).

#### Data Analyses

The gender difference was explored by multivariate analyses of covariance with gender as the grouping variable and age as a covariate to control for the influence of age. Internal consistency was measured using Cronbach's alpha for the ITQ and other instruments, where a Cronbach's alpha above 0.7 was deemed acceptable, and 0.80 or more was recommended (Polit and Beck, 2013). We defined the number of factors to be extracted through parallel analysis, which compared the progressive eigenvalues from the given data matrix to those of a simulated data matrix using random data of the same size (Horn, 1965). According to recommendations, 500 random datasets were generated, and the 95th percentiles of the eigenvalues from these random datasets were compared to those of the actual dataset. If the eigenvalue of the actual data was greater than the corresponding eigenvalue of the random data, the number of factors was retained (O'Connor, 2000; Timmerman and Lorenzo-Seva, 2011). Before the exploratory factor analysis (EFA), Bartlett's Test of Sphericity was applied to ensure the non-randomness of the correlation matrix (*p*-value should be < 0.05), whereas the Kaiser-Meyer-Olkin (KMO) measure of sampling adequacy was calculated to ensure that the matrices were suitable for analysis (should be > 0.60) (Tabachnick and Fidell, 2007). We used the robust weighted least squares adjusted for mean and variance estimator and a polychoric correlation matrix that was appropriate for ordinal data.

We employed the unweighted least squares method with geomin rotation, and the hierarchical Schmid–Leiman solution to identify the factor structure of the scale (Schmid and Leiman, 1957). Confirmatory factor analysis (CFA) was conducted to examine the fit of the original and proposed models. As the variables were nonparametric, we performed the CFA by using a robust estimator (the maximum likelihood estimation with robust standard errors and a mean- and variance-adjusted, MLMV) that appropriately corrects for the standard errors of the parameters. To evaluate the model fit, we calculated the following indices and adopted the generally recommended criteria: chi-square (χ^2^), degree of freedom (df), χ^2^/df (2.0–5.0), root mean square error of approximation (RMSEA, <0.06), comparative fit index (CFI, >0.95), and Tucker–Lewis Index (TLI, >0.95) (Hu and Bentler, 1999; Tabachnick and Fidell, 2007). The intercorrelations among the specific factors and the general factor were assessed using Spearman's correlation (Spearman's *r* = 0.00–0.19 implies very weak, 0.20–0.39 implies weak, 0.40–0.59 implies moderate, 0.60–0.79 implies strong 0.80–1.00 implies very strong).

SPSS 17.0, FACTOR (Lorenzo-Seva and Ferrando, 2019) and Mplus Version 8 (Muthén and Muthén, 2017) were used for all data analysis.

## Results

### Descriptive Statistics and Internal Consistency of the ITQ Scale and Subscales

Descriptive data and sex differences (as shown in Table 2) for ITQ subscales are presented in Table 3. The Cronbach's alpha coefficients varied from 0.59 (Focus) to 0.79 (ITQ Total) based on the total sample, which demonstrates acceptable internal consistency. We checked the corrected item-total correlation of the relatively low alpha coefficients of focus subscales and found that the item-total correlation ranged from 0.21 to 0.36. Men scored higher than women on the *ITQ total* score and the following subscales: *Involvement* and *Games*. Women scored higher than men on the *Focus* subscale.

**Table 2:** Internal consistency reliabilities (α), means, standard deviations (SD), and gender differences on the ITQ-18 scale and subscales.

**Scale and subscales**	**Number of items**	**α**	**Women**		**Men**		**F (1.778)**	***P*** **-value**	**Partial η2**
			**Mean**	**SD**	**Mean**	**SD**			
Involvement	7	0.78	31.85	5.93	33.43	6.77	4.58	0.033	0.006
Focus	7	0.59	27.27	8.06	25.87	8.95	11.28	0.001	0.014
Games	2	0.70	3.27	2.11	6.26	3.87	192.80	0.000	0.199
ITQ Total	18	0.80	71.01	13.76	75.26	17.63	15.64	0.000	0.020

*The F tests and effect sizes (partial η2) were for multivariate analyses of covariance with gender as the grouping variable, and age was included as a covariate to control for the influence of age (Male n = 192; Female n = 588)*.

**Table 3:** Results of the CFA of the ITQ and ITQ-10.

	***χ2***	***χ2/DF***	***RMSEA***	***CFI***	***TLI***
**Based on the original 18-item**				
Three correlated factors	1,428.44	13.60	0.127	0.607	0.550
Unidimensional structure	1,405.25	13.51	0.127	0.613	0.554
**Based on the EFA results (10 items)**				
Two-correlated factors	286.09	8.41	0.068	0.866	0.822
Unidimensional structure	585.76	16.73	0.142	0.706	0.622
Bifactor solution	87.39	3.49	0.057	0.967	0.940

*CFA, Confirmatory Factor Analysis; EFA, Exploratory Factor Analysis; DF, Degrees of Freedom; RMSEA, Root Mean Square Error of Approximation; CFI, Comparative Fit Index; TLI, Tucker-Lewis Index*.

#### The Dimensionality of the Immersive Tendency

In the first step, we tested the original three-factor model of the 18-item scale by CFA (Table 3). As the fit indices were lower than acceptable, EFA was conducted to investigate alternative factor structures. In the next step, we performed an EFA. Kaiser-Myer-Olkin measures of sampling adequacy were good (KMO: 0.80, Bartlett's Test of Sphericity *p* < 0.001), which suggested that the data were suitable for factor analyses. To determine the number of factors to be extracted, we performed a parallel analysis by creating 500 randomly generated correlation matrices. The eigenvalues were 5.07, 2.19, and 1.68, which accounted for 49.8% of the total variance (28.2, 12.2, and 9.4%; respectively). Consequently, the first factor explained a relatively large proportion of the variance, indicating that all items may have belonged to a single dimension. However, the parallel analysis suggested the extraction of two factors because they accounted for a sufficient amount of variance. Robust Unweighted Least Squares (RULS) factor extraction with Robust Promin rotation (Lorenzo-Seva and Ferrando, 2019) was conducted in the total sample. The results of factor analysis are reported in Table 3. To delineate the underlying factor structure optimally, communalities (h^2^) were examined and items with low values (i.e., < 0.35) were eliminated from subsequent analyses (Costello and Osborne, 2005). This resulted in the elimination of eight items. The EFA-derived two-factor solution (gray cells) was validated by CFA. To find the most appropriate model, we tested both the unidimensional and two-dimensional models (correlated factors and bifactor solution). As shown in Table 4, relative model fit comparisons reveal that the bifactor solution fits better than the two-correlated factor solution. A bifactor model would specify a single general (primary) immersion factor reflecting the common variance of all items and two narrower groups (specific) factors reflecting the variance of their assigned items. Thus, each item simultaneously loads on the general factor and its assigned group factor. These three factors are all first-order ones set orthogonally to each other. The general factor reflects the primary factor independent of the group factors, whereas each specific factor reflects the unique factor of interest after controlling (partialling out) the general factor.

**Table 4:** The factor structure of the 18-item version of the ITQ.

**ITQ items**	**Original subscale**	**F1 focus**	**F2 involvement**	**h^2^**
1. Do you easily become deeply involved in movies or TV dramas?	Focus	0.18	0.51	0.35
2. Do you ever become so involved in a TV program or book that people have problems getting your attention?	Involvement	0.08	0.61	0.41
3. How mentally alert do you feel at present?	Focus	0.63	−0.27	0.36
4. Do you ever become so involved in a movie that you are unaware of things happening around you?	Involvement	0.03	0.74	0.56
5. How frequently do you find yourself closely identifying with the characters in a storyline?	Involvement	0.09	0.58	0.37
6. Do you ever become so involved in a video game that it is like you are inside the game rather than moving a joystick and watching the screen?	Games	−0.09	0.56	0.29
7. How physically fit do you feel today?	Focus	0.60	−0.19	0.35
8. How good are you at blocking out external distractions when you are involved in something?	Focus	0.65	−0.02	0.42
9. While watching sports, do you ever become so involved in the game that you react as if you were one of the players?		0.16	0.32	0.16
10. Do you ever become so involved in a daydream that you are unaware of things happening around you?	Involvement	−0.10	0.72	0.48
11. Do you ever have dreams that are so real that you feel disoriented when you wake up?	Involvement	−0.18	0.59	0.32
12. While playing sports, do you become so involved in the game that you lose track of time?	Focus	0.24	0.36	0.24
13. How well do you concentrate on enjoyable activities?		0.59	0.08	0.38
14. How often do you play arcades or video games? (The term “often” should be taken to mean every day or every 2 days, on average.)	Games	−0.09	0.35	0.11
15. Have you ever got excited during a chase or fight scene on TV or in the movies?	Focus	0.07	0.55	0.34
16. Have you ever got scared by something happening on a TV show or in a movie?	Involvement	−0.06	0.58	0.31
17. Have you ever remained apprehensive or fearful long after watching a scary movie?	Involvement	−0.17	0.45	0.19
18. Do you ever get so involved in doing something that you lose all track of time?	Focus	0.14	0.57	0.40

*h^2^: communalities*.

#### The External Validity of the ITQ-10

As seen in Table 5, the ITQ-10 scale and subscales had a high positive correlation with the original ITQ scales. As expected, the ITQ-10 focus scale was correlated positively with all adaptive personality traits (e.g., SCCS, MHC-SF), and negatively with maladaptive features (e.g., ASI, SPQ-BR, PID-5-BF, BFAS, PIUQ). The involvement subscale of the ITQ seems to be a strong predictive factor for the potential clinical problems (e.g., ASI, SPQ-BR, negative affectivity, disinhibition, psychoticism, BFAS, PIUQ).

**Table 5:** Short for ITQ-10 and their correlations with other trait predispositions and behavioral habits.

	**Focus**	**Involvement**	**ITQ total**
**Original ITQ scales**			
Focus	0.71^**^		
Involvement		0.89^**^	
ITQ total			0.89^**^
**External validity scales**			
TAS	0.051	0.45^**^	0.41^**^
SCCS	0.50^**^	−0.38^**^	−0.19^**^
MHC-SF	0.48^**^	−0.06	0.16^**^
ASI	−0.24^**^	0.22^**^	0.08*
SPQ-BR	−0.27^**^	0.32^**^	0.15^**^
**PID-5-BF**			
Negative affects (PID-5-BF)	−0.31^**^	0.23^**^	0.06
Detachment (PID-5-BF)	−0.33^**^	0.00	−0.14^**^
Antagonism (PID-5-BF)	−0.17^**^	0.05	−0.02
Disinhibition (PID-5-BF)	−0.33^**^	0.23^**^	0.05
Psychoticism (PID-5-BF)	−0.27^**^	0.33^**^	0.17^**^
BFAS	−0.18^**^	0.25^**^	0.13^**^
PIUQ	−0.24^**^	0.33^**^	0.17^**^

*TAS, Tellegen Absorption Scale; SCCS, Self-Concept Clarity Scale; MHC-SF, Mental Health Continuum Short Form; ASI, Anxiety Sensitivity Index; SPQ-BR, Schizotypal Personality Questionnaire-Brief Revisited; PID-5-BF, Personality Inventory for DSM-5*.

## Discussion

In the first part of this study, we introduced the ITQ-10, which is a short form of the original version of the ITQ. Two associated factors have been defined that behave differently during the use of social media practice. The focused attention factor is associated with adaptive traits, and the involvement tendency factor is associated with maladaptive personality traits and digital media using habits. The second part of the presented data showed detailed personality trait patterns in which the content of the presented ITQ factors can be explored. The most essential elements of social adaptation are life satisfaction, self-gratification, a positive and supportive, rewarding social environment, self-clarity, and self-coherence (Keyes, 2013). Our ITQ-10 results showed that using digitally mediated communication channels helps to build social health and a supportive environment in people who can distinguish between self-relevant and self-irrelevant events and have low anxiety, negative affect, antagonism, disinhibition psychoticism, detachment scores, and low scores in problematic Internet use and Facebook dependence. These adaptive trait tendencies are strongly associated with aware and controlled focused attention capabilities. On the other hand, although involvement tendencies *via* the absorption trait play a role in the emotional and cognitive immersion and the intensive reaction of the visual scenes, most frequently associated with maladaptive traits and habits. The involvement tendencies are associated with higher anxiety, schizotypal traits, negative effects, disinhibition, psychoticism scores, problematic internet usage, and Facebook dependency.

Traditional learning theories have interpreted human behavior as a consequence of directly experienced response effects. From a social cognitive perspective, learning is a continuous complementary interaction between an individual's behavior and the conditions controlling it. In the learning process, understanding social experiences and critical processing serve as determining parameters (Bandura, 1986). The motivational resources, open-mindedness for storing, actively engaging, and well-determined goals are true for the usage of authentic and digital media devices, and for communication devices: reading a book, watching television, surfing the Internet, using Facebook, learning, or working in a digital or virtual environment (Baranyai et al., 2021). Emotional and motivational effects triggered by the source of information are more emphasized if the recipient is more open to the content (Tagg, 2015). There are individual differences in terms of openness, reliving, emotional involvement, and immersion in the available media or digital environments (Park et al., 2009; Orchard and Fullwood, 2010).

We explored personal immersion during the consumption of popular entertainment and the use of digital communication devices. We found that the short form of ITQ involves two correlating but relatively different forms of immersion. The *involvement* factors relate to the self and environment boundary weakness that can be considered a partly maladaptive behavioral function and is related to a higher degree of Facebook addiction, problematic Internet use, negative affectivity, disinhibition, and schizotypal personality trait. Our data support other research results indicating that the mentioned traits and habits play a maladaptive role in the social and psychological wellbeing and the development of the personality (Burke et al., 2010; Cicei, 2012; Garcia and Sikström, 2014; Cicero, 2017; Hargitai et al., 2020). Furthermore, our results are consonant with the suggestion that the uncertain self-coherence and the associated loneliness, low emotional accessibility, and the frequently associated problematic internet usage habit manifests in trait and behavioral patterns that are similar to the schizotypal personality characteristics (Király et al., 2015; Truzoli et al., 2016; Salvarli and Griffiths, 2019).

However, the *attention focus* factor refers to higher mental control, adaptive potential, and the capability to discriminate between self and non-self-related events; clear self-definition, higher wellbeing, and mental and physical health; a lower degree of personality disorders and schizotypal traits; and strong and adaptive mental control in Facebook and problematic Internet use. There are a lot of different aspects that must be satisfied when it comes to the choice of research tool. These results are consonant with other examination reports (Glicksohn and Berkovich-Ohana, 2012; Keyes, 2013; Parsons et al., 2015), and support Kardefelt-Winther (2014) suggestion on the origin of problematic media use, namely, the maladaptive, and dependent social media usage is a compensating maneuver to avoid the schizotypal malfunctioning and to alleviate the social handicaps.

When one is immersed in or fully focused on an event, they can lose their connection to the real world, and the events around them can become detached. In this state, a person could be immersed in a book, a song, a video, a game, or a conversation on Facebook, and may lose interest in everyday issues. According to the authors of the first significant questionnaire that measures immersion tendencies, the person “melts” into the story or environment (Witmer and Singer, 1998). A masterfully narrated story, an artistic composition, and realistic visual and auditory parameters are basic conditions for immersion into an experience (Slater, 2009). Previous definitions suggest that the immersion experience not only describes a personal competency but also the technical structure of the environment and a realistically portrayed event. A sophisticated user is immersed in a well-crafted artificial environment (Toma and Hancock, 2013). With advancements in technology, the description of an immersion experience has also diversified over the last few years. The event horizon of a person who is immersed in a digital environment is wide. The connection between a person and the digital environment is more interactive in the present. The user can freely choose to reveal or hide from the public. The users can freely choose to reveal themselves or remain hidden from the public presence. This duality—the will to obtain current and new knowledge and the hypothetical possibility of taking possession of an endless amount of Internet knowledge—could result in oppressive internal conflicts among social media users (Tosun, 2012; Ujhelyi, 2014).

In summary, wellbeing and personal social and psychological growth capacity, a positive emotional tendency to constrict safe social networks and partnerships, personal autonomy, and goal-oriented behavior with skills and talent to adequate executive capability are the foundations of social adaptation (Keyes, 2013). Considering our results, it can be stated that besides the physically real world numerous other facilities are available to have adequate knowledge and experience of the environment. The computer-generated and digitally mediated environment are considered a socially mediated transmitter between the individual and the physical and social environment. The rate of the expedience for immersion depends on the controllability of attention allocation that can discriminate between self-relevant and self-irrelevant information. The mental focusing ability contributes to the form of a healthy environment and the sustainability of the self. However, they need to account for the disadvantage used of social media and the dependency on the digital world. The incoherent self provides a rich ground for the development of maladaptive behaviors and attitudes, and as a result, some people are unable to break free from their digital prison. The problem solution is given, let to work on the immersion but at the same time, you develop a coherent self and guide your attention consciously focusing on your long-term goals.

## Conclusion

The presented study examined the socially adaptive or socially maladaptive personal tendencies when individuals use social media such as the internet or Facebook. The digitally mediated social media is an essential agent in modern life, but its function for the individual is different. Usually, social media usage is socially beneficial, but in persons with vulnerability to social deficiency the usage of these communication channels, and devices may induce disadvantageous consequences such as social isolation, dependencies, cognitive disorders, and delays in social development. The presented study points to the importance of validated measurement methods in the exploration of the advantageous or disadvantageous effects of frequent maladaptive digital media usage. The results of the statistical analyses of the ITQ highlighted that the same psychological mechanism, immersion, in the digitally mediated environment can play a different role in an individual with vulnerability in the construction and keeping of social interaction disorders. The immersion and engagement with the content of the digitally mediated messages are beneficial contributions to the social development, but only when the individual has a clear self-concept and conscious mental control over attention allocation to switch between relevant and irrelevant messages and information. The short form of the ITQ is adequate to assess the relationship between the self-referred and environment-dependent psychological functions. These results point to an efficient investigation target to understand the role of self-coherence and self-control in digitally mediated social media usage in physically real and digitally mediated environments.

## Data Availability Statement

The raw data supporting the conclusions of this article will be made available by the authors, without undue reservation.

## Ethics Statement

The studies involving human participants were reviewed and approved by University of Pécs, No. 6732 PTE/2017. The patients/participants provided their written informed consent to participate in this study.

## Author Contributions

SR and JK: study conception and design, drafting of the manuscript, critical revision. RH, AL, OA, and IT: analysis and interpretation of the data, and drafting of the manuscript. EH: acquisition of data, analysis, and interpretation of data, and drafting of the manuscript. All authors read and approved the final manuscript.

## Funding

This study was supported by the Hungarian National Research, Development, and Innovation Office (Grant Number: NKFI K 120334) and Károli Gáspár University of Reformed Church (Personality and Health Psychology Department, Grant Number: 20692B800).

## Conflict of Interest

The authors declare that the research was conducted in the absence of any commercial or financial relationships that could be construed as a potential conflict of interest.

## Publisher's Note

All claims expressed in this article are solely those of the authors and do not necessarily represent those of their affiliated organizations, or those of the publisher, the editors and the reviewers. Any product that may be evaluated in this article, or claim that may be made by its manufacturer, is not guaranteed or endorsed by the publisher.
